# SLR: a scaffolding algorithm based on long reads and contig classification

**DOI:** 10.1186/s12859-019-3114-9

**Published:** 2019-10-30

**Authors:** Junwei Luo, Mengna Lyu, Ranran Chen, Xiaohong Zhang, Huimin Luo, Chaokun Yan

**Affiliations:** 1 College of Computer Science and Technology, Henan Polytechnic University, Jiaozuo, 454000 China; 2 School of Computer and Information Engineering, Henan University, Kaifeng, 475001 China

**Keywords:** Scaffolding, Genome assembly, Sequence analysis, Pacific biosciences, Oxford Nanopore

## Abstract

**Background:**

Scaffolding is an important step in genome assembly that orders and orients the contigs produced by assemblers. However, repetitive regions in contigs usually prevent scaffolding from producing accurate results. How to solve the problem of repetitive regions has received a great deal of attention. In the past few years, long reads sequenced by third-generation sequencing technologies (Pacific Biosciences and Oxford Nanopore) have been demonstrated to be useful for sequencing repetitive regions in genomes. Although some stand-alone scaffolding algorithms based on long reads have been presented, scaffolding still requires a new strategy to take full advantage of the characteristics of long reads.

**Results:**

Here, we present a new scaffolding algorithm based on long reads and contig classification (SLR). Through the alignment information of long reads and contigs, SLR classifies the contigs into unique contigs and ambiguous contigs for addressing the problem of repetitive regions. Next, SLR uses only unique contigs to produce draft scaffolds. Then, SLR inserts the ambiguous contigs into the draft scaffolds and produces the final scaffolds. We compare SLR to three popular scaffolding tools by using long read datasets sequenced with Pacific Biosciences and Oxford Nanopore technologies. The experimental results show that SLR can produce better results in terms of accuracy and completeness. The open-source code of SLR is available at https://github.com/luojunwei/SLR.

**Conclusion:**

In this paper, we describes SLR, which is designed to scaffold contigs using long reads. We conclude that SLR can improve the completeness of genome assembly.

## Background

With the increasing availability of third-generation sequencing technologies, which include Single-Molecule Real-Time (SMRT) technology from Pacific Biosciences and Nanopore-based technology from Oxford Nanopore, many biological applications have been greatly improved. Compared with second-generation sequencing technologies, third-generation sequencing technologies produce longer reads with a higher sequencing error rate [1]. In the field of de novo genome assembly, a large number of assembly tools based on third-generation sequencing technologies have been presented to resolve the most prominent problem: repetitive regions. However, producing a complete and accurate assembly is still a challenging task. Scaffolding is an important step in the pipeline of genome assembly, and aims to orient and order contigs [2, 3]. Scaffolding generates scaffolds consisting of sequence fragments including oriented and ordered contigs. The gap between two adjacent contigs in a scaffold is filled with ’N’ characters. Scaffolding can significantly increase the continuity of assembly results and benefit downstream analyses such as those of gene order, comparative or functional genomics and patterns of recombination [4].

According to the kind of reads used for scaffolding, existing scaffolding tools generally fall into the following three categories:

(i) Using paired reads for scaffolding. The insert size of paired reads can reach a few thousands bases, so this technique can partially resolve the problem of repetitive regions. Such scaffolding tools, such as OPERA [5], SSPACE [6], BESST [7], ScaffMatch [8], SCARPA [9], ScaffoldScaffolder [10], and BOSS [11], usually use greedy heuristic algorithms to generate scaffolds based on a scaffold graph, in which a vertex denotes a contig and an edge represents the existence of paired reads that can be separately aligned to the two corresponding contigs. However, because the length of reads from second-generation technologies is commonly only a few hundred bases, the reads can usually be aligned with two or more positions in the contigs. Moreover, the region between the paired reads is unknown, and there are sequencing errors in the reads. Some spurious edges are usually introduced into a scaffold graph, which complicates the scaffolding task. Obtaining more accurate and contiguous scaffolding results based on paired reads is a difficult task.

(ii) Using long reads for scaffolding. This kind of scaffolding tool usually aligns the long reads against contigs first and then finds contigs that can be aligned with the same long read. Then, these tools use the local alignment result to infer the global order and orientation of contigs. For instance, SSPACE-LongRead [12] first aligns whole long reads with contigs using the alignment tool BLASR [13]. Next, contig pairs and multi-contig linkage information are obtained and used to order and orient the contigs and generate scaffolds. LINKS [14] does not align the whole long reads to the contigs; it first extracts the k-mer pairs in an interval from long reads. Afterwards, these k-mer pairs are aligned to the contigs, and the alignment results are used to link the contigs. Finally, LINKS selects a neighbour of a contig as its correct neighbour based on the number of links. SMSC [15] first aligns the long reads to the contigs using either Nucmer [16] or BLASR and then constructs a breakpoint graph in which a vertex is a contig and an edge is added to indicate a long read bridging two vertices. It transforms the scaffolding problem to a maximum alternating path coverage problem in the breakpoint graph and resolves this problem using a 2-approximation algorithm. RAILS [17] scaffolds contigs with long reads using the scaffolding engine originally developed for SSAKE [18] and LINKS. Based on the sequencing coverage of each contig, npScarf [19] classifies contigs into unique contigs and repetitive contigs. npScarf first bridges the unique contigs and generates scaffolds based on a greedy strategy and then fills the gaps by repetitive contigs. However, most contig sets used for scaffolding do not include information on sequencing coverage, which limits the application of npScarf.

(iii) Using optical mapping data or Hi-C data for scaffolding. Optical mapping data can serve as a unique "fingerprint" or "barcode" for genome sequences. By comparing optical mapping data with a restriction enzyme map of the contigs, the order and orientation of contigs can be inferred. Supernova [20], Architect [21], ARCS [22] and fragScaff [23] attempt to find pairs of contigs based on linked reads. The problem with using optical mapping data is that a barcode used to locate contigs may have many different alignment positions, which usually causes contradictions between contigs. Hi-C data are commonly sequenced by paired-end sequencing. The paired reads come from the interacting fragments between genomic loci that are nearby in three-dimensional space but may be separated by many nucleotides in the linear genome. Scaffolding using Hi-C data is the most challenging method, as the genomic distance between a given Hi-C-based read pair is highly variable and may span a few kilobases to megabases without any direct indication of the true distance [1].

Although some scaffolding tools based on long reads have made great progress, two primary issues still require more attention. (i) Scaffold graph construction: In a scaffold graph, each vertex refers to a contig, and an edge is created between two vertices if the two contigs can be aligned with the same long read. Due to the repetitive regions in contigs and the high sequencing error rate of long reads, the scaffold graph usually becomes very complicated, which has negative effects on the later scaffolding steps. Hence, simplifying the scaffold graph is a significant goal for scaffolding. (ii) Edge weighting: In the scaffold graph, most current methods prefer to weight each edge by the number of long reads that can be aligned with two vertices simultaneously. However, the length of the alignment between a long read and a contig can reflect the confidence level of the alignment, which is usually ignored by existing methods.

When a long read links the two flanking regions of a repetitive region, the problem of the repetitive region can be resolved because the order and orientation of the two flanking regions can be obtained directly. Moreover, a repetitive region can usually be aligned with more than one long read, and their 5’-end (or 3’-end) neighbour regions are not the same. After aligning the long reads against the contigs, we can identify whether contigs are repetitive based on their aligniment positions in the long reads. When constructing a scaffold graph, it is difficult to avoid spurious edges introduced by repetitive contigs and sequencing errors. We can identify spurious edges by detecting orientation and position contradictions in the scaffold graph [10, 11]. Using only non-repetitive contigs to construct a scaffold graph not only simplifies the complexity of the scaffold graph but also improves the accuracy of spurious edge detection.

In this paper, we present a scaffolding algorithm based on long reads and contig classification (SLR), which utilizes two new strategies to address the two issues above. For issue (i), SLR classifies the contigs into unique contigs and ambiguous contigs. SLR utilizes the unique contigs to construct a scaffold graph, which can decrease the complexity of the scaffold graph and simplify the following scaffolding steps. For issue (ii), SLR uses the alignment length to weight each edge in the scaffold graph. Moreover, SLR employs linear programming to detect and remove the contradictions in the scaffold graph, which guarantees that the scaffold graph includes only simple paths.

Based on these two new strategies, SLR determines the orientations and orders of the contigs. In experiments, SLR is compared with three popular scaffolding tools by scaffolding five long-read datasets with Pacific Biosciences and Oxford Nanopore technologies. The experimental results show that SLR produces better results in terms of accuracy and completion for most datasets.

## Results

To evaluate the performance of SLR, we compared SLR with three popular scaffolding tools based on long reads, namely, SSPACE-LongRead (SSPACE-LR), LINKS and npScarf.

### Datasets and metrics

Contig and long-read datasets for *Escherichia coli (E. coli)*, *Saccharomyces cerevisiae W303 (S. cerevisiae)*, and *Human chromosome X (Chr X)* were utilized as input for all tools. *E. coli* and *S. cerevisiae* include two different long-read datasets sequenced with Pacific Biosciences and Oxford Nanopore technologies and consist of two different contig sets assembled by different assemblers. The long reads for *Chr X* are from Pacific Biosciences. The details of the long-read datasets are shown in Table 1. The contig sets, which were evaluated by QUAST [24], are shown in Table 2. Then, these contig sets and long-read sets form nine datasets, shown in Table 3, were used for scaffolding, and each dataset included one contig set and one long-read set. We named the nine datasets as *E. coli*_1_SMRT, *E. coli*_2_SMRT, *S. cerevisiae*_1_SMRT, *S. cerevisiae*_2_SMRT, *Chr X*_1_SMRT, *E. coli*_1_ONT, *E. coli*_2_ONT, *S. cerevisiae*_1_ONT, and *S. cerevisiae*_2_ONT.

**Table 1:** Details of long-read datasets

	*E. coli*		*S. cerevisiae*		*Chr X*
Genome size(Mbp)	4.6		12.1		155.2
Sequencing technology	SMRT	Nanopore	SMRT	Nanopore	SMRT
Read N50(bp)	5,189	8,484	6,794	8,608	11,030
Number of reads	81,737	20,750	594,243	410,344	1,135,220
Name	*E. coli*_SMRT	*E. coli*_ONT	*S. cerevisiae*_SMRT	*S. cerevisiae*_ONT	*Chr X*_SMRT

**Table 2:** Details of contig sets

Contig set	Count	Errors	Genome	Mismatches	Indels	Largest	NA50	NGA50
			Fraction(%)			alignment		
*E. coli*_1	182	2	99.363	1.32	0.37	315,628	106,208	106,208
*E. coli*_2	167	7	99.351	2.28	0.11	360,084	164,044	164,044
*S. cerevisiae*_1	3179	35	96.688	79.03	8.42	233,103	47,994	52,239
*S. cerevisiae*_2	6953	53	96.687	85.54	8.76	250,180	49,258	54,160
*Chr X*_1	8623	41	97.037	2.40	1.28	793,618	76,506	71,372

The contig set about *E. coli*_1 and *S. cerevisiae*_1 are provided by [29], the contig set about *E. coli*_2 and *S. cerevisiae*_2 are provided by [30], and the contig set about *Chr X*_1 are provided by [31]

**Table 3:** Datasets used for scaffolding and evaluations for SLR and SLR1

Dataset		Genome	Contig set	Long read set		Misassemblies			NGA50	
						SLR	SLR1		SLR	SLR1
*E. coli*_1_SMRT		*E. coli*	*E. coli*_1	*E. coli*_SMRT		4	12		723,879	295,999
*E. coli*_2_SMRT		*E. coli*	*E. coli*_2	*E. coli*_SMRT		10	11		565,864	197,175
*S. cerevisiae*_1_SMRT		*S. cerevisiae*	*S. cerevisiae*_1	*S. cerevisiae*_SMRT		52	57		374,744	232,712
*S. cerevisiae*_2_SMRT		*S. cerevisiae*	*S. cerevisiae*_2	*S. cerevisiae*_SMRT		71	67		270,402	201,922
*Chr X*_1_SMRT		*Chr X*	*Chr X*_1	*Chr X*_SMRT		83	82		2,390,483	2,165,615
*E. coli*_1_ONT		*E. coli*	*E. coli*_1	*E. coli*_ONT		4	8		2,927,247	674,408
*E. coli*_2_ONT		*E. coli*	*E. coli*_2	*E. coli*_ONT		9	14		733,062	361,345
*S. cerevisiae*_1_ONT		*S. cerevisiae*	*S. cerevisiae*_1	*S. cerevisiae*_ONT		46	66		374,835	244,417
*S. cerevisiae*_2_ONT		*S. cerevisiae*	*S. cerevisiae*_2	*S. cerevisiae*_ONT		68	85		270,362	201,066

Each dataset includes one contig set and one long-read set, and corresponds to one genome.

QUAST aligns the contigs (or scaffolds) to the reference genome and obtains some metrics. NG50 is the length of the longest contig (or scaffold) such that all the contigs (or scaffolds) of that length or longer cover at least half of the reference genome. N50 is the length of the longest contig (or scaffold) such that all the contigs (or scaffolds) of that length or longer cover at least half of the length of all contigs (or scaffolds). Misassemblies (Errors) is the number of positions (breakpoints) in the contigs or scaffolds in which errors (Translocation, Inversion, Relocation) occur. NGA50 is the NG50 of contigs or scaffolds after they have been broken at every breakpoint. Genome Fraction is the percentage of aligned bases in the reference genome. Usually, Misassemblies can represent the accuracy of the scaffolding result, and NGA50 and NA50 can reflect the completion and continuity of the scaffolding result. In the experiments below, we used QUAST to evaluate the scaffolding results for SSPACE-LR, LINKS, npScarf and SLR.

### Evaluations on nine datasets

**Fig. 1:**
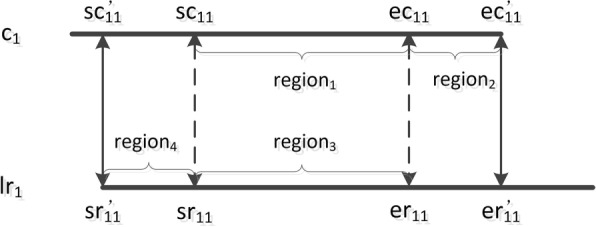
Nine figures plotting NGA50 vs Misassemblies. The results of SLR usually can be found in the top-left corner, which can illustrate the advantage of SLR

The long-read sets about first five datasets are obtained by SMRT technology. And, the long-read sets about last four datasets are obtained by Nanopore technology. All the scaffolding tools were run on these nine datasets, and detailed evaluation results from QUAST are shown in Additional file 1: Tables S1 and S2. Because NGA50 and Misassemblies are two important metrics for evaluating scaffolding tools, we show NGA50 vs Misassemblies in Fig. 1. The best scaffolding result can found in the top-left corner of each figure. Except in Fig. 1(b) and Fig. 1(i), SLR is in the top-left corner throughout Fig. 1, which indicates that SLR has lower Misassemblies and a higher NGA50. Although npScarf performs better in Fig. 1(b) and Fig. 1(i), the performance of SLR is close to it.

### Running time and peak memory

Due to the high error rate in long reads, aligning long reads with contigs usually takes a long time. LINKS selects k-mer pairs from the long reads to link the contigs, which avoids long read alignment. However, LINKS requires more memory to store the k-mer pairs. As shown in Table 4, we find that LINKS consumes less time and more memory. SLR and npScarf have similar time consumption, because both use BWA-MEM [25] to align long reads against contigs. In all experiments, npScarf allocates a large memory despite the size of the dataset. When extracting alignment information from the BAM file, SLR keeps the alignment of one long read in memory and produces a local scaffold that is saved on the hard disk. After processing one long read, SLR processes the next long read, which can reduce the memory requirement. Compared with other tools, SSPACE-LR and SLR require less memory for scaffolding.

**Table 4:** Running time and peak memory

Dataset		Running time					Peak memory (G)			
		SSPACE-LR	LINKS	npScarf	SLR		SSPACE-LR	LINKS	npScarf	SLR
*E. coli*_1_SMRT		41m42s	1m42s	26m34s	26m58s		1.00	6.23	10.28	1.04
*E. coli*_2_SMRT		42m12s	1m42s	26m46s	28m17s		1.00	6.22	10.28	1.17
*S. cerevisiae*_1_SMRT		880m22s	38m26s	929m36s	907m23s		3.96	93.27	10.28	1.86
*S. cerevisiae*_2_SMRT		1162m28s	40m35s	1012m27s	957m59s		3.96	93.3	10.28	3.60
*Chr X*_1_SMRT		8413m53s	41m2s	6617m53s	7782m13s		12.56	114.2	12.82	3.98
*E. coli*_1_ONT		46m25s	2m22s	28m6s	26m57s		1.02	8.60	10.28	1.05
*E. coli*_2_ONT		47m57s	2m25s	28m17s	28m42s		1.01	8.60	10.28	1.11
*S. cerevisiae*_1_ONT		676m26s	41m20s	962m51s	1001m25s		3.43	117.97	10.28	1.84
*S. cerevisiae*_2_ONT		830m48s	40m54s	1046m16s	1051m53s		3.52	117.99	10.28	3.47

### Effectiveness of contig classification

To verify the effectiveness of the contig classification method presented in this paper, we removed the step of contig classification from SLR and this new algorithm was named SLR1. Then, we benchmarked SLR with SLR1 on all datasets. The scaffolding results for SLR and SLR1 are shown in Table 3. We can see that SLR performs better than SLR1 in terms of Misassemblies and NGA50. Therefore, we can prove that our proposed contig classification method is effective.

**Fig. 2:**
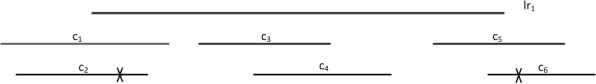
Contig classification combines with SSPACE-LR and LINKS

Next, we combined the contig classification method with other scaffolding tools. SLR classified each contig set into a unique contig set and an ambiguous contig set. We first ran SSPACE-LR and LINKS on the unique contig set, generating some scaffolds. Then, we inserted the ambiguous contigs into the scaffolds. For this purpose, we should determine the order and orientation of the unique contigs in these scaffolds. BWA-MEM is used to align the unique contigs against these scaffolds. Only if a unique contig is completely aligned in a scaffold, the corresponding alignment is retained. Then, we can obtain the order and orientation of the unique contigs in these scaffolds. The final scaffolding results is shown in Fig. 2. SSPACE-LR-CC represents the method based on SSPACE-LR combined with contig classification. LINKS-CC represents the method based on LINKS combined with contig classification. According to Fig. 2, we find that SSPACE-LR-CC and LINKS-CC outperformed SSPACE-LR and LINKS in NGA50. This further confirms the effectiveness of the method of contig classification.

Compared with SLR, SSPACE-LR-CC outperformed SLR in NGA50 for *E. coli*_2_SMRT and *Chr X*_1_SMRT. For the remaining seven datasets, SLR performed better than SSPACE-LR-CC in NGA50. SLR performed better than LINKS-CC in NGA50 for all datasets. Meanwhile, SLR outperformed SSPACE-LR-CC and LINKS-CC in Misassemblies for all datasets.

The detailed evaluation results are provided in Additional file 1. Note that, because npScarf makes sequence consensus between contigs and long reads, it is difficult to identify the order of the unique contigs in the scaffolds. We did not use npScarf in the this experiment.

### Evaluation using a repeat-aware evaluation framework

We also used a repeat-aware evaluation framework [26] to evaluate the performance of SSPACE-LR, LINKS, npScarf and SLR. For each original contig set, by aligning contigs with the reference genome, this framework splits contigs in misassembly events, and extracts repetitive sub-contig from original contigs. Then, it outputs a new contig set. The framework records the number of correct links, which is the number of correct contig joins. After a scaffolding tool runs on this new contig set and a long-read set, the framework computes the number of correctly predicted links. Therefore, we can compute precision, recall and F1-score for the scaffolding results. For the contig set about *Chr X*, the framework ran for more than one week and gave no new contig set. Hence, we processed only the remaining original contig sets. So, there are eight new datasets used for this experiment, which are named *E. coli*_1_SMRT_R, *E. coli*_2_SMRT_R, *S. cerevisiae*_1_SMRT_R, *S. cerevisiae*_2_SMRT_R, *E. coli*_1_ONT_R, *E. coli*_2_ONT_R, *S. cerevisiae*_1_ONT_R, and *S. cerevisiae*_2_ONT_R. The detailed evaluation results provided by the framework are shown in Additional file 1: Tables S9 and S10.

**Fig. 3:**
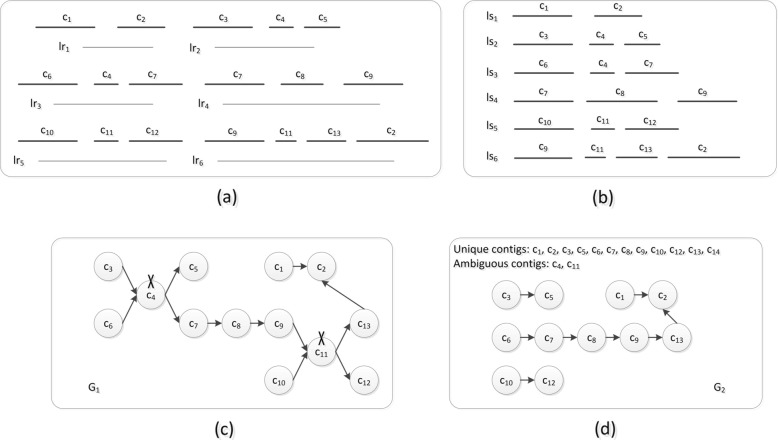
NGA50 for datasets produced by repeat-aware evaluation framework

In addition, for these new datasets, we also evaluated the scaffolding results by QUAST, which are shown in Fig. 3. According to Fig. 3, SLR achieved the best NGA50 values for all the datasets. This experiment shows that SLR can identify repetitive contigs and overcome the problem of repeating regions.

## Discussion

npScarf utilizes sequencing coverage to classify contigs. However, most contigs used for scaffolding do not include information about sequencing coverage, which limits the application of npScarf. SLR can classify contigs without any additional information about the contig set. SSPACE-LR uses a greedy heuristic strategy to determine the neighbour of a contig based on the number of long reads that can be aligned. LINKS uses a strategy similar to that of SSPACE-LR to determine the neighbours by counting the number of k-mer pairs between two contigs. These two tools have difficulty identifying the correct neighbours when encountering complex repetitive regions.

## Conclusion

With the development of third-generation high-throughput sequencing technologies, scaffolding methods based on long reads have undergone substantial improvement. A scaffold graph is the basis for inferring the orders and orientations of contigs. However, the problems introduced by repetitive regions and sequencing errors pose challenges in the process of constructing scaffold graphs. In this paper, we presented a novel scaffolder, SLR, for determining the orientations and orders of contigs based on long reads and contig classification. SLR employs a new contig classification procedure to overcome the problems associated with repetitive regions in scaffolding. SLR first produces local scaffolds based on the alignment between long reads and contigs. A local scaffold corresponds to a long read and the contigs that can be aligned with it. SLR classifies contigs into unique and ambiguous contigs based on local scaffolds. A scaffold graph including only unique contigs is constructed; this process can simplify the scaffold graph and improve the accuracy of detecting and removing contradictions. Experiments were conducted that included long reads obtained with SMRT-based and Nanopore-based technologies. The experimental results illustrated that SLR is superior in terms of continuity and accuracy. For larger genomes, such as the complete human genome, however, SLR is difficult to scale due to its long run time.

## Method

A contig set *C* and a long read set *LR* are used as input data. The algorithm is composed of four steps: (i) producing local scaffolds; (ii) classifying contigs; (iii) constructing a scaffold graph; and (iv) generating scaffolds. In the first step, the alignment tool BWA-MEM is used to align *LR* against *C*. For each long read and set of contigs that can be aligned with it, SLR determines the orders and orientations of the contigs and forms a local scaffold. In the second step, SLR classifies the contigs into unique contigs and ambiguous contigs based on their positions in the local scaffolds. In the third step, SLR constructs a scaffold graph based on unique contigs and then detects and removes the contradictions in the scaffold graph. In the fourth step, SLR extracts the simple paths from the scaffold graph to yield a draft scaffold set. Next, SLR inserts the ambiguous contigs into the draft scaffolds. The details of each step are described below. Note that the long reads whose lengths are longer than *L*_*r*_ and the contigs whose lengthes are longer than *L*_*c*_ are used by SLR. *L*_*r*_ and *L*_*c*_ are two parameters that can be defined by users. In addition, if a contig is completely contained in other contigs, SLR will ignore it in the following scaffolding steps.

### Producing local scaffolds

SLR utilizes BWA-MEM to align *LR* against *C*, and the SAM file is converted to a BAM file by Bamtools [27]. Due to the high sequencing error rate in long reads, the alignment positions are usually different from the real positions. With the following method, SLR first revises the alignment positions and obtains reliable alignments.

**Fig. 4:**
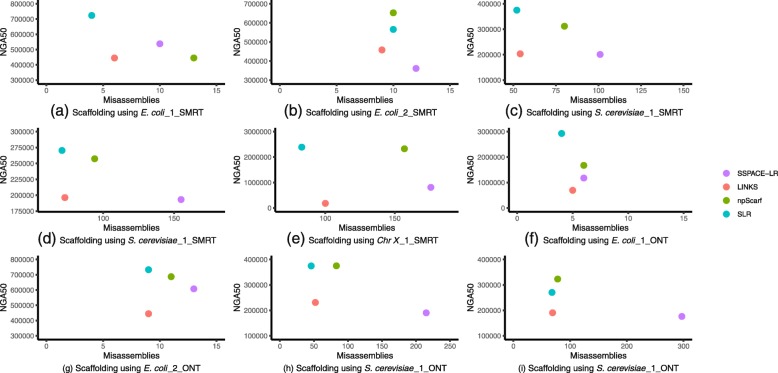
An example of alignment position revision. For an alignment given by the alignment tool, the region [ *sr*_11_, *er*_11_] (*region*_3_) in the long read *lr*_1_ is aligned with the region [ *sc*_11_, *ec*_11_] (*region*_1_) in the contig *c*_1_. Because *sr*_11_<*sc*_11_ and *LEN*(*lr*_1_)−*er*_11_>*LEN*(*c*_1_)−*ec*_11_, it means the region [0, *sr*_11_] (*region*_4_) in *lr*_1_ is not aligned with *c*_1_, and the region [ *ec*_11_,*LEN*(*c*_1_)−1] (*region*_2_) is not aligned with *lr*_1_. However, when *lr*_1_ is truely aligned with *c*_1_ and the alignment is reliable, *region*_4_ should be aligned with the region [ *sc*_11_−*sr*_11_, *sc*_11_] in *c*_1_, and *region*_2_ should be aligned with the region [ *er*_11_, *er*_11_+*LEN*(*c*_1_)−*ec*_11_]. Because of the high sequencing error rate in long reads, the alignment tool usually does not provide accurate alignment start and end positions. Then, SLR sets *sc*11′=*sc*_11_−*sr*_11_, *sr*11′=0, *ec*11′=*LEN*(*c*_1_)−1 and *er*11′=*er*_11_+*LEN*(*c*_1_)−*ec*_11_. When the alignment is reliable, the region [ *sc*11′, *ec*11′] in *c*_1_ is aligned with the region [ *sr*11′, *er*11′] in *lr*_1_

For an alignment between the *j*-th long read *lr*_*j*_ and the *i*-th contig *c*_*i*_, we assume that the region [ *sr*_*ij*_, *er*_*ij*_] in *lr*_*j*_ is aligned with the region [ *sc*_*ij*_, *ec*_*ij*_] in *c*_*i*_. If *sr*_*ij*_<*sc*_*ij*_, *srij*′ = 0 and *scij*′ = *sc*_*ij*_ - *sr*_*ij*_, else *scij*′ = 0 and *srij*′ = *sr*_*ij*_ - *sc*_*ij*_. If *LEN*(*lr*_*j*_)−*er*_*ij*_>*LEN*(*c*_*i*_)−*ec*_*ij*_, *erij*′=*er*_*ij*_+*LEN*(*c*_*i*_)−*ec*_*ij*_ and *ecij*′ = *LEN*(*c*_*i*_)−1, else *ecij*′=*ec*_*ij*_+*LEN*(*lr*_*j*_)−*er*_*ij*_ and *erij*′=*LEN*(*lr*_*j*_)−1. *LEN*(*lr*_*j*_) and *LEN*(*c*_*i*_) are the lengths of *lr*_*j*_ and *c*_*i*_ respectively. [ *srij*′, *erij*′] and [ *scij*′, *ecij*′] are the real alignment regions. An example of the revision is shown in Fig. 4. After revision, the alignment will be reliable if the following hold: i) The mapping quality is higher than *s*_*m*_ (a threshold with a default 20); ii) both the values *er*_*ij*_−*sr*_*ij*_ and *ec*_*ij*_−*sc*_*ij*_ are greater than *l*_*m*_ (a threshold with a default 100); iii) for each of *sr*_*ij*_, *er*_*ij*_, *sc*_*ij*_ and *ec*_*ij*_, the difference between its original position and its revised position is smaller than *α* (a threshold with a default 150). SLR retains only reliable alignments.

**Fig. 5:**
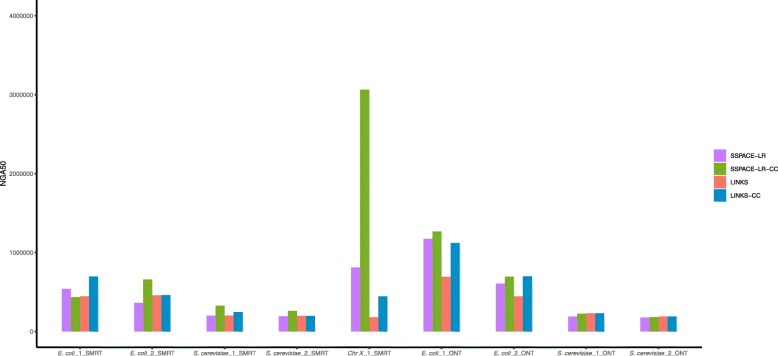
There are six contigs (*c*_1_,*c*_2_,*c*_3_,*c*_4_,*c*_5_,*andc*_6_) that can be aligned with the long read *lr*_1_. Because *c*_1_ and *c*_2_ are simultaneously aligned with the left end of *lr*_1_, SLR retains only contig *c*_1_ which has the greatest alignment length, and deletes the alignment information between *c*_2_ and *lr*_1_. Because *c*_5_ and *c*_6_ have been simultaneously aligned with the right end of *lr*_1_, we keep only *c*_5_, and delete the alignment information between *c*_6_ and *lr*_1_. Finally, SLR determines the orders and orientations of *c*_1_, *c*_3_, *c*_4_ and *c*_5_, which form a local scaffold

A local scaffold is composed of ordered and oriented contigs that can be aligned with the same long read. The *i*-th local scaffold *ls*_*i*_ is represented by the vertex sequence *s*_*i*1_,*s*_*i*2_,...*s*_*im*_, where *m* is the number of contigs in the *i*-th local scaffold. *s*_*ij*_ is represented by a four-tuple (*sc*_*ij*_,*sco*_*ij*_,*scg*_*ij*_,*scl*_*ij*_). *sc*_*ij*_ refers to the *j*-th contig in *ls*_*i*_. *sco*_*ij*_ denotes the alignment orientation between the contig and the long read. *sco*_*ij*_=1 represents forward alignment. *sco*_*ij*_=0 represents reverse alignment. *scg*_*ij*_ denotes the gap distance between *sc*_*ij*_ and *sc*_*i*(*j*+1)_. In particular, the gap distance of the last vertex is zero. *scl*_*ij*_ is the alignment length between *sc*_*ij*_ and the long read. Note that if there are two or more contigs aligned with the same end of the long read, SLR keeps only the contig that has the greast alignment length. An example is shown in Fig. 5.

The contig *sc*_*ij*_ is in the middle position of *ls*_*i*_ if 1<*j*<*m*. *sc*_*ij*_ and *sc*_*i*(*j*+1)_ are adjacent in *ls*_*i*_. If *sco*_*ij*_=1, *sc*_*i*(*j*−1)_(*j*>1) is the 5’-end neighbour contig of *sc*_*ij*_, and *sc*_*i*(*j*+1)_(*j*<*m*) is the 3’-end neighbour contig of *sc*_*ij*_. If *sco*_*ij*_=0, *sc*_*i*(*j*−1)_(*j*>1) is the 3’-end neighbour contig of *sc*_*ij*_, and *sc*_*i*(*j*+1)_(*j*<*m*) is the 5’-end neighbour contig of *sc*_*ij*_.

In this step, SLR finally obtains a local scaffold set *LS*. Due to the high sequencing error rate, a contig may not be aligned with the long read that connects its left and right neighbour contigs. To resolve this problem, SLR deletes some local scaffolds. For example, the local scaffold *ls*_1_ is (*A*,*C*), and the local scaffold *ls*_2_ is (*B*,*C*). If the sum of *LEN*(*B*) and the gap distance between *B* and *C* in *ls*_2_ is smaller than the gap distance between *A* and *C* in *ls*_1_ and there exists a local scaffold (*A*,*B*,*C*), SLR removes *ls*_1_.

### Classifying contigs

Repetitive regions are the critical problem in the process of scaffolding. When constructing a scaffold graph, the 5’-end (or 3’-end) of a repetitive contig can usually be linked with two or more other contigs, which complicates the scaffold graph. Because repetitive contigs commonly emerge in many different local scaffolds, they have two or more distinct 5’-end (or 3’-end) neighbour contigs. When a contig is not in the middle position of any local scaffold, no long read can span the contig to link its two neighbour contigs, and this contig is usually a long unique contig. Although the contig has multiple 5’-end or 3’-end neighbour contigs, SLR uses contradiction removal step to identify its correct neighbour contigs. Hence, SLR can identify whether a contig is unique based on its positions in the local scaffolds.

To reduce the negative effects of short repetitive contigs, SLR considers a contig whose length is shorter than *L*_*ca*_ (a threshold that can be set by users) to be an ambiguous contig. These short contigs are temporally ignored in the local scaffolds. Next, the contigs longer than *L*_*ca*_ are classified using the following method.

SLR identifies a contig as ambiguous if the following hold: i) The contig is in the middle position of one or more local scaffolds and ii) the number of 5’-end (or 3’-end) neighbour contigs of the contig is greater than one.

**Fig. 6:**
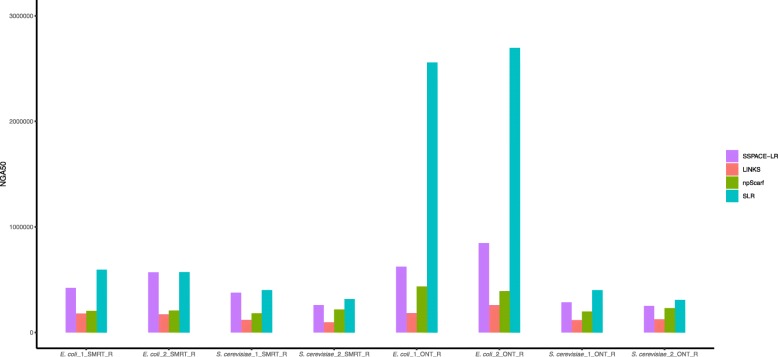
(a) There are six long reads: *lr*_1_, *lr*_2_, *lr*_3_, *lr*_4_, *lr*_5_, and *lr*_6_. The contigs *c*_1_ and *c*_2_ are aligned with *lr*_1_. *c*_3_, *c*_4_ and *c*_5_ are aligned with *lr*_2_. *c*_6_, *c*_4_ and *c*_7_ are aligned with *lr*_3_. *c*_7_, *c*_8_ and *c*_9_ are aligned with *lr*_4_. *c*_10_, *c*_11_ and *c*_12_ are aligned with *lr*_5_. *c*_9_, *c*_11_, *c*_13_ and *c*_2_ are aligned with *lr*_6_. We assume that all these alignments are forward, and all contigs are longer than *L*_*ca*_. (b) Based on the alignment result described in (a), SLR obtains six local scaffolds: *ls*_1_, *ls*_2_, *ls*_3_, *ls*_4_, *ls*_5_, and *ls*_6_. (c) The scaffold graph *G*_1_ is built using all contigs. We find that *G*_1_ is complicated. (d) Based on the contig classification method described in Section 2.2, the contigs can be divided into two categories. Because *c*_4_ is located in the middle position of *ls*_2_ and *ls*_3_ and has two distinct 3’-end neighbours and two distinct 5’-end neighbour contigs, it is identified as an ambiguous contig. *c*_11_ is also an ambiguous contig. The remaining contigs are identified as unique contigs. The scaffold graph *G*_2_ is built based on unique contigs and is thus less complicated than *G*_1_

After all ambiguous contigs have been identified, the remaining contigs are considered unique contigs. In this way, the contigs are classified into unique contigs and ambiguous contigs by SLR. An example of such contig classification is shown in Fig. 6.

### Constructing a scaffold graph

A scaffold graph *G* is represented by a vertex set *V* and an edge set *E*. A vertex *v*_*i*_ corresponds to a contig *c*_*i*_. An edge *e*_*ij*_ is denoted by a five-tuple (*v*_*i*_,*v*_*j*_,*o*_*ij*_,*g*_*ij*_,*w*_*ij*_). Two vertices *v*_*i*_ and *v*_*j*_ are connected by *e*_*ij*_. *g*_*ij*_ is the gap distance between *v*_*i*_ and *v*_*j*_. *o*_*ij*_ is the relative orientation of *v*_*i*_ and *v*_*j*_. There are four types of relative orientation between *v*_*i*_ and *v*_*j*_: (i) the 3’-end of *v*_*i*_ is connected to the 5’-end of *v*_*j*_; (ii) the 5’-end of *v*_*i*_ is connected to the 3’-end of *v*_*j*_; (iii) the 5’-end of *v*_*i*_ is connected to the 5’-end of *v*_*j*_, and (iv) the 3’-end of *v*_*i*_ is connected to the 3’-end of *v*_*j*_. For types (i) and (ii), *v*_*i*_ and *v*_*j*_ are on the same strand. For the other two types, *v*_*i*_ and *v*_*j*_ are on the opposite strands. *w*_*ij*_ is the weight of the edge, which reflects its confidence.

Neglecting the ambiguous contigs and constructing scaffold graph *G* with only unique contigs will significantly simplify *G* and reduce the difficulties in inferring the orders and orientations of the unique contigs. Therefore, all unique contigs make up the vertex set *V*. Below, we describe how to create the edge set *E*. The superiority of constructing a scaffold graph using unique contigs is illustrated in Fig. 6.

#### Adding edges to the scaffold graph

First, SLR ignores the ambiguous contigs in all local scaffolds; therefore, some non-adjacent unique contigs may become adjacent in one local scaffold. Assume that the *i*-th local scaffold *ls*_*i*_(*s*_*i*1_,*s*_*i*2_,...*s*_*im*_) in *LS* includes two adjacent unique contigs *sc*_*ip*_ and *sc*_*is*_. If one or more ambiguous contigs exist between *sc*_*ip*_ and *sc*_*is*_, the gap distance between *sc*_*ip*_ and *sc*_*is*_ is re-calculated by formula (1); otherwise, it is equal to *scg*_*ip*_. Here, *GD*(*sc*_*ip*_,*sc*_*is*_,*lr*_*i*_) represents the gap distance between *sc*_*ip*_ and *sc*_*is*_ in *ls*_*i*_. Moreover, SLR can obtain a weight value, which is the minimum value of *scl*_*ip*_ and *scl*_*is*_. The weight value can be used to evaluate the confidence level of the relation between *sc*_*ip*_ and *sc*_*is*_. As the weight value becomes larger, the order of the two unique contigs becomes more reliable.

1 $$ GD(sc_{ip}, sc_{is}, lr_{i}) = \sum\limits_{j = p}^{s - 1} {scg_{ij}} + \sum\limits_{j = p + 1}^{s - 1} {LEN(sc_{ij})}  $$

We assume that *sc*_*ip*_ is represented by *c*_*a*_ and that *sc*_*is*_ is represented by *c*_*b*_. For *c*_*a*_ and *c*_*b*_, SLR selects all local scaffolds in which *c*_*a*_ and *c*_*b*_ are adjacent. Next, SLR determines the relative orientation of the gap distance between and weight of *c*_*a*_ and *c*_*b*_ based on these local scaffolds. For two unique contigs, the relative order and orientation should be unique. If different values of *o*_*ab*_ are obtained from the local scaffolds, SLR keeps only the local scaffold set *LS*_*ab*_ for which the value of *o*_*ab*_ is the same, and the number of elements in *LS*_*ab*_ is the largest. The gap distance between *c*_*a*_ and *c*_*b*_ is calculated according to formula (2). In addition, we can obtain a weight value for each local scaffold in *LS*_*ab*_. The final weight of *c*_*a*_ and *c*_*b*_ (denoted *w*_*ab*_) can be obtained by seeking the maximum weight value obtained by the local scaffolds in *LS*_*ab*_. Then, SLR adds an edge *e*_*ab*_ to *G*.

2 $$ g_{ab} = \frac{{\sum\limits_{i = 1}^{n} {GD(c_{a},c_{b},ls_{i})}}}{n}  $$

in which *n* is the number of elements in *LS*_*ab*_, and *ls*_*i*_∈*LS*_*ab*_.

After processing all pairs of unique contigs in *LS*, a draft scaffold graph *G* can be constructed by SLR for the subsequent steps.

#### Removing contradictions

Due to sequencing errors in long reads and complex repetitive regions, the scaffold graph *G* may still contain some spurious edges. Detecting and removing the spurious edges in *G* can be viewed as detecting and removing the orientation and position contradictions [10, 11]. BOSS utilizes an iterative strategy to detect and remove contradictions. BOSS first constructs a sub-graph that includes only edges with a high weight. Next, it iteratively adds the remaining edges to the sub-graph from high to low weight. Each iteration includes a sub-graph, and BOSS builds two linear programming models [28] to solve orientation and position contradictions in the sub-graph. SLR utilizes a revised method based on BOSS to remove contradictions. The difference in SLR compared to BOSS is that SLR adds all edges to the sub-graph in the first iteration. Hence, SLR completes contradiction removal within one iteration, while BOSS requires several iterations. The methods of building the linear programming model of BOSS and SLR are the same, as described below.

First, SLR detects and deletes orientation contradictions. For the edge *e*_*ij*_∈*G*, if *o*_*i*_≠*o*_*j*_, SLR constructs constraint Eq. (3). If *o*_*i*_=*o*_*j*_, SLR constructs constraint Eq. (4).

3 $$ \eta_{ij} < = o_{i} + o_{j} < = 2 - \eta_{ij}  $$

4 $$ \eta_{ij} - 1 < = o_{i} - o_{j} < = 1 - \eta_{ij}  $$

in which *η*_*ij*_∈{0,1} is a variable that represents whether *e*_*ij*_ is spurious. 0_*i*_∈{0,1} is also a variable that denotes the orientation of *v*_*i*_. The objective function is $MAX(\sum \ {(w_{ij}*\eta _{ij})})$.

Second, SLR detects and deletes position contradictions. For the edge *e*_*ij*_∈*G*, SLR constructs constraint Eq. (5).

5 $$ L(\phi_{ij} - 1) < = p_{j} - p_{i} - len(c_{i}) - gd_{ij} < = L(1 - \phi_{ij})  $$

in which *p*_*i*_ is a variable that represents the assigned position of *v*_*i*_. *ϕ*_*ij*_ is a slack variable in the range [0,1] that reflects the consistency between *g*_*ij*_ and |*p*_*j*_−*p*_*i*_|. The objective function is $MAX(\sum \ {(w_{ij}*\phi _{ij})})$. For an edge, if the gap distance computed by the assigned position is far from the original one, the edge is deemed spurious one, and then SLR deletes it from *G*.

After eliminating the orientation and position contradictions, if there are two or more edges linking the same end of a vertex, SLR keeps only the edge with the highest weight and removes the others. Consequently, the scaffold graph *G* contains only simple paths.

### Generating scaffolds

Each simple path in *G* refers to a scaffold, and SLR selects all simple paths and constructs a draft scaffold set. For any two adjacent vertices in the draft scaffold, SLR scans the local scaffold set *LS* again and finds local scaffolds that contain them. If ambiguous contigs exist between these vertices in a local scaffold, these ordered and oriented ambiguous contigs correspond to a path. If there are two or more different paths, SLR selects the one with the greatest number of local scaffolds that support it and then inserts it between the two vertices. Note that an ambiguous contig may occur two or more times in the scaffolds.

Next, SLR selects local scaffolds that contain the first contig of a scaffold. SLR constructs a scaffold graph based on these local scaffolds. If a simple path starts from the first contig in the scaffold graph, it is merged with the head of the scaffold. In the same way, SLR extends the tail of the scaffold. Once the first *t* contigs of a scaffold are the same as the last *t* contigs of another scaffold (*t* is a threshold set by users), SLR will merge them together to form a new scaffold. In the same way, SLR will reverse a scaffold and detect whether it can be merged with other scaffolds. Finally, SLR outputs the scaffolds as the final result.

## Supplementary information

**Additional file 1** It includes seven sections: (i) Datasets; (ii) Command lines; (iii) Scaffolding results about nine datasets; (iv) Different values of *L*_*ca*_ for scaffolding; (v) Scaffolding results about SLR1 and SLR2; (vi) SSPACE-LR and LINKS combined with contig classification method; (vii) Scaffolding results based on repeat-aware evaluation framework.

## Data Availability

All datasets used in this paper and command lines for all scaffolding tools are provided in Additional file 1.

## Abbreviations

*Chr X*: *Human chromosome X*
*E. coli*: *Escherichia coli*
*S. cerevisiae*: *Saccharomyces cerevisiae W303*
SLR: Scaffolding algorithm based on Long Reads and contig classification
SMRT: Single-Molecule Real-Time
SSPACE-LR: SSPACE-LongRead
