# Correction to: SLR: a scaffolding algorithm based on long reads and contig classification

**DOI:** 10.1186/s12859-020-3362-8

**Published:** 2020-02-10

**Authors:** Junwei Luo, Mengna Lyu, Ranran Chen, Xiaohong Zhang, Huimin Luo, Chaokun Yan

**Affiliations:** 1 College of Computer Science and Technology, Henan Polytechnic University, Jiaozuo, 454000 China; 2 School of Computer and Information Engineering, Henan University, Kaifeng, 475001 China

**Correction to: BMC Bioinformatics (2019) 20:539**

**https://doi.org/10.1186/s12859-019-3114-9**

Following publication of the original article [1], the author reported that there is an error in the original article;

1. The figures’ order in HTML and PDF are incorrect.
  In the original article incorrect Fig. 1 is the correct Fig. 4.
  In the original article incorrect Fig. 2 is the correct Fig. 5.
  In the original article incorrect Fig. 3 is the correct Fig. 6.
  In the original article incorrect Fig. 4 is the correct Fig. 1.
  In the original article incorrect Fig. 5 is the correct Fig. 2.
  In the original article incorrect Fig. 6 is the correct Fig. 3.

**Fig. 1:**
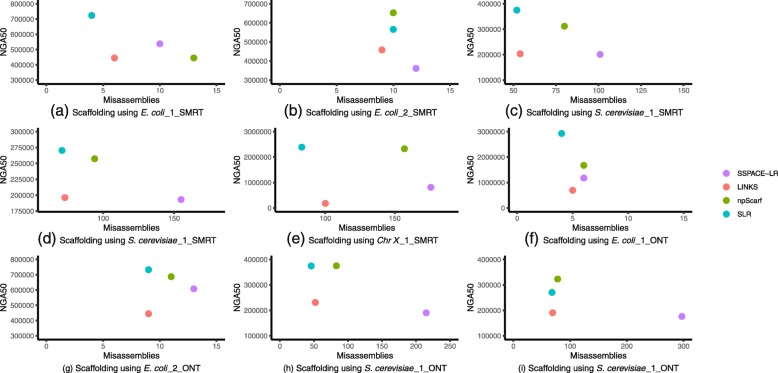
Nine figures plotting NGA50 vs Misassemblies. The results of SLR usually can be found in the top-left corner, which can illustrate the advantage of SLR

**Fig. 2:**
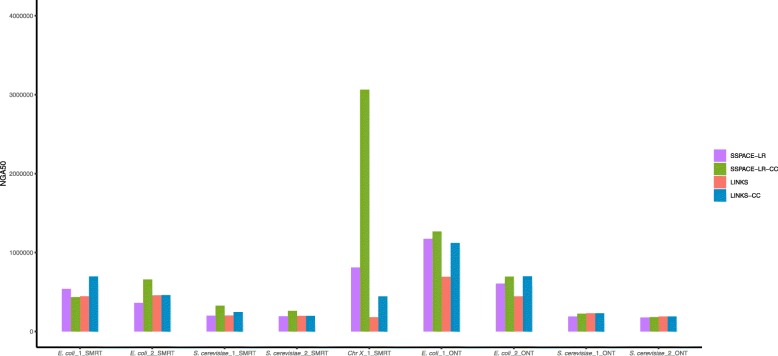
Contig classification combines with SSPACE-LR and LINKS

**Fig. 3:**
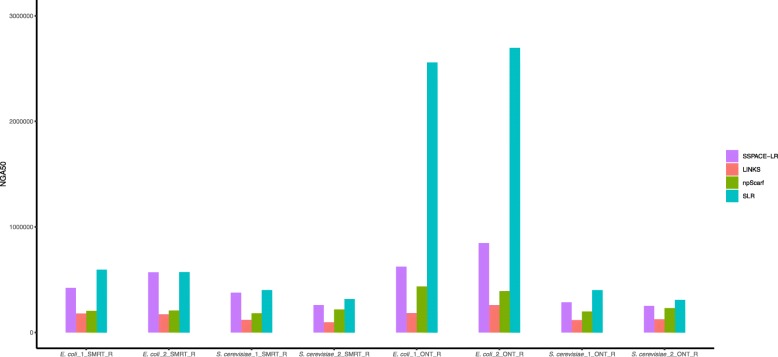
NGA50 for datasets produced by repeat-aware evaluation framework

**Fig. 4:**
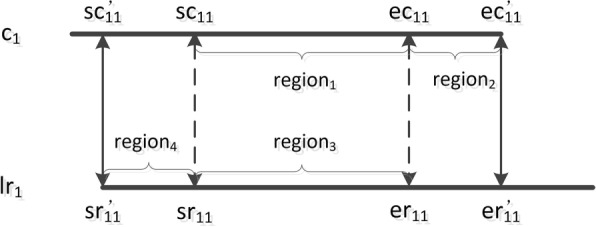
An example of alignment position revision. For an alignment given by the alignment tool, the region [*sr*_11_, *er*_11_] (*region*_3_) in the long read *lr*_1_ is aligned with the region [*sc*_11_, *ec*_11_] (*region*_1_) in the contig *c*_1_. Because *sr*_11_ < *sc*_11_ and *LEN*(*lr*_1_) − *er*_11_ > *LEN*(*c*_1_) − *ec*_11_, it means the region [0, *sr*_11_] (*region*_4_) in *lr*_1_ is not aligned with *c*_1_, and the region [*ec*_11_,*LEN*(*c*_1_) − 1] (*region*_2_) is not aligned with *lr*_1_. However, when *lr*_1_ is truely aligned with *c*_1_ and the alignment is reliable, *region*_4_ should be aligned with the region [*sc*_11_ − *sr*_11_, *sc*_11_] in *c*_1_, and *region*_2_ should be aligned with the region [*er*_11_, *er*_11_ + *LEN*(*c*_1_) − *ec*_11_]. Because of the high sequencing error rate in long reads, the alignment tool usually does not provide accurate alignment start and end positions. Then, SLR sets *sc*11′ = *sc*_11_ − *sr*_11_, *sr*11′ = 0, *ec*11′ = *LEN*(*c*_1_) − 1 and *er*11′ = *er*_11_ + *LEN*(*c*_1_) − *ec*_11_. When the alignment is reliable, the region [*sc*11′, *ec*11′] in *c*_1_ is aligned with the region [*sr*11′, *er*11′] in *lr*_1_

**Fig. 5:**
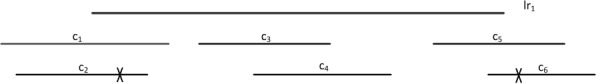
There are six contigs (*c*_1_,*c*_2_,*c*_3_,*c*_4_,*c*_5_,*andc*_6_) that can be aligned with the long read *lr*_1_. Because *c*_1_ and *c*_2_ are simultaneously aligned with the left end of *lr*_1_, SLR retains only contig *c*_1_ which has the greatest alignment length, and deletes the alignment information between *c*_2_ and *lr*_1_. Because *c*_5_ and *c*_6_ have been simultaneously aligned with the right end of *lr*_1_, we keep only *c*_5_, and delete the alignment information between *c*_6_ and *lr*_1_. Finally, SLR determines the orders and orientations of *c*_1_, *c*_3_, *c*_4_ and *c*_5_, which form a local scaffold

**Fig. 6:**
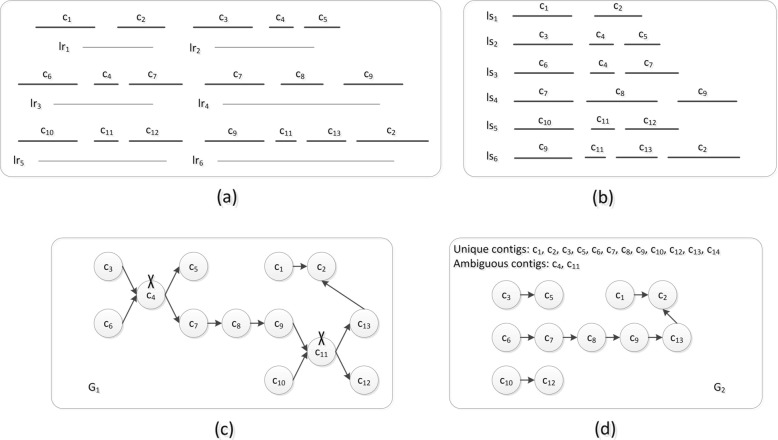
(a) There are six long reads: *lr*_1_, *lr*_2_, *lr*_3_, *lr*_4_, *lr*_5_, and *lr*_6_. The contigs *c*_1_ and *c*_2_ are aligned with *lr*_1_. *c*_3_, *c*_4_ and *c*_5_ are aligned with *lr*_2_. *c*_6_, *c*_4_ and *c*_7_ are aligned with *lr*_3_. *c*_7_, *c*_8_ and *c*_9_ are aligned with *lr*_4_. *c*_10_, *c*_11_ and *c*_12_ are aligned with *lr*_5_. *c*_9_, *c*_11_, *c*_13_ and *c*_2_ are aligned with *lr*_6_. We assume that all these alignments are forward, and all contigs are longer than *L*_*ca*_. (b) Based on the alignment result described in (a), SLR obtains six local scaffolds: *ls*_1_, *ls*_2_, *ls*_3_, *ls*_4_, *ls*_5_, and *ls*_6_. (c) The scaffold graph *G*_1_ is built using all contigs. We find that *G*_1_ is complicated. (d) Based on the contig classification method described in Section 2.2, the contigs can be divided into two categories. Because *c*_4_ is located in the middle position of *ls*_2_ and *ls*_3_ and has two distinct 3′-end neighbours and two distinct 5′-end neighbour contigs, it is identified as an ambiguous contig. *c*_11_ is also an ambiguous contig. The remaining contigs are identified as unique contigs. The scaffold graph *G*_2_ is built based on unique contigs and is thus less complicated than *G*_1_

In this correction article the figures are shown correct.
