# Supplementary material for: SLR: a scaffolding algorithm based on long reads and contig classification
Source: BMC Bioinformatics. 2019 Oct 30;20:539. doi: 10.1186/s12859-019-3114-9 (PMC6820941; doi:10.1186/s12859-019-3114-9)
Supplement: Supplementary file 1 — Additional file 1 It includes seven sections: (i) Datasets; (ii) Command lines; (iii) Scaffolding results about nine datasets; (iv) Different values of Lca for scaffolding; (v) Scaffolding results about SLR1 and SLR2; (vi) SSPACE-LR and LINKS combined with contig classification method; (vii) Scaffolding results based on repeat-aware evaluation framework. [file 12859_2019_3114_MOESM1_ESM.doc]

**Additional file 1**

**1. Datasets**

**2. Command lines**

**3. Scaffolding results about nine datasets**

**4. Different values of L_ca_ for scaffolding**

**5. Scaffolding results about SLR1 and SLR2**

**6. SSPACE-LR and LINKS combined with contig classification method**

**7. Scaffolding results based on repeat-aware evaluation framework**

**1. Datasets**

All datasets used in this paper can be download from the following web sites.

***E. coli*_1, the first contig set about *E. coli*:** http://labshare.cshl.edu/shares/schatzlab/www-data/ectools/ecoli/ecoli_illumina.fa.gz

***E. coli*_2, the second contig set about *E. coli*:** http://labshare.cshl.edu/shares/schatzlab/www-data/nanocorr/2015.07.07/ecoli_Miseq_Assembly.fa.gz

***S. cerevisiae*_1, the first contig set about *S. cerevisiae:*** http://labshare.cshl.edu/shares/schatzlab/www-data/ectools/w303/w303_illumina.fa.gz

***S. cerevisiae*_2, the second contig set about *S. cerevisiae*:** http://labshare.cshl.edu/shares/schatzlab/www-data/nanocorr/2015.07.07/W303_Miseq_Assembly.fa.gz

***Chr X*_1, the contig set about *Chromosome X*:** http://www.fishbrowser.org/software/LR_Gapcloser/

***E. coli*_SMRT, the long-read set of *E. coli* based on SMRT sequencing technology:**

https://trace.ncbi.nlm.nih.gov/Traces/sra/?run=SRR801650

***S. cerevisiae*_SMRT, the long-read set of *S. cerevisiae* W303 based on SMRT sequencing technology:**

http://labshare.cshl.edu/shares/schatzlab/www-data/ectools/w303/Pacbio.fasta.gz

***E. coli*_ONT, the long-read set of *E. coli* based on Oxford Nanopore sequencing technology:**

ftp://penguin.genomics.cn/pub/10.5524/100001_101000/100102/Ecoli_R7_CombinedFasta.tgz (NormalTwoDirectionReads.fasta)

***S. cerevisiae*_ONT, the long-read set of *S. cerevisiae* W303 based on Oxford Nanopore sequencing technology:**

http://labshare.cshl.edu/shares/schatzlab/www-data/nanocorr/2015.07.07/W303_ONT_Raw_reads.fa.gz

***Chr X*_SMRT, the long-read set of *Human Chromosome X* based on SMRT sequencing technology:**

http://www.fishbrowser.org/software/LR_Gapcloser/

**2. Command lines**

All experiments are run in a computer with Ubuntu System, Intel(R) Xeon(R) CPU E5-2650, 10 cores and 128G memory. The command lines for the four scaffolding tools are shown as follow:

**SSPACE-LR, version 1-1:**

perl SSPACE-LongRead.pl -c <contig-set-file> -p <long-read-set-file>

**LINKS, version 1.8.6:**

./LINKS -t 3 -f <contig-set-file> -s <long-read-set.fof>. -b <output-directory>

(long-read-set.fof is the file stores the address of the long read set file.)

For dataset about Chrx, ./LINKS -t 25 -f <contig-set-file> -s <long-read-set.fof>. -b <output-directory>

**npScarf:**

jsa.seq.sort -r -n --input <contig-se-filet> --output <sort-contig-set-file>

bwa index sort-contig-set-file

bwa mem -t 8 -k11 -W20 -r10 -A1 -B1 -O1 -E1 -L0 -a -Y <sort-contig-set-file> <long-read-set-file> > aligning.sam

jsa.np.npscarf -seq <sort-contig-set-file> -input <aligning.sam> -format sam

**SLR, version 1.0:**

bwa index contig-set-file

bwa mem -a contig-set-file contig-set-file > align-self.sam

samtools view -Sb align-self.sam > align-self.bam

bwa mem -t 8 -k11 -W20 -r10 -A1 -B1 -O1 -E1 -L0 -a -Y <contig-set-file> <long-read-set-file> > aligning.sam

samtools view -Sb aligning.sam > aligning.bam

SLR -c <contig-set-file> -r <aligning.bam> -d < align-self.bam > -p <output-directory>

**3. Scaffolding results about nine datasets**

In the main text, we give a figure about NGA50 vs Misassemblies. The detailed results evaluated by QUAST are shown in Table S1 and Table S2.

Table S1. Evaluation results based on SMRT datasets

| Data set | Tool | Count | MA^1^ | Genome Fraction | Mismatches^2^ | Indels^3^ | Largest alignment | NG50 | NGA50 |
| --- | --- | --- | --- | --- | --- | --- | --- | --- | --- |
| *E.coli_*1_SMRT | SSPACE-LR | 102 | 10 | 99.550 | 2.12 | 1.21 | 952,193 | 1,084,156 | 538,319 |
|  | LINKS | 111 | 6 | 99.351 | 1.71 | 1.02 | 855,618 | 506,227 | 445,266 |
|  | npscarf | 96 | 13 | 99.561 | 4.63 | 2.94 | 693,218 | 668,859 | 444,955 |
|  | SLR | 83 | 4 | 99.873 | 2.07 | 3.39 | 1,011,630 | 723,879 | 723,879 |
| *E.coli*_2_SMRT | SSPACE-LR | 124 | 12 | 99.364 | 2.25 | 0.54 | 693,087 | 661,478 | 361,326 |
|  | LINKS | 127 | 9 | 99.354 | 2.23 | 0.41 | 855,943 | 654,326 | 458,412 |
|  | npscarf | 126 | 10 | 99.443 | 3.05 | 1.23 | 698,773 | 656,803 | 652,603 |
|  | SLR | 118 | 10 | 99.598 | 2.99 | 1.54 | 855,361 | 1,130,614 | 565,864 |
| *S.cerevisiae*_1_SMRT | SSPACE-LR | 2237 | 101 | 97.209 | 84.61 | 13.51 | 576,123 | 245,251 | 200,813 |
|  | LINKS | 2800 | 54 | 96.734 | 78.77 | 10.25 | 741,980 | 262,335 | 203,642 |
|  | npscarf | 2755 | 80 | 97.437 | 110.24 | 21.42 | 1,069,953 | 799,048 | 312,122 |
|  | SLR | 2784 | 52 | 97.250 | 95.79 | 12.71 | 1,079,981 | 736,056 | 374,744 |
| *S.cerevisiae*_2_SMRT | SSPACE-LR | 5260 | 155 | 97.421 | 95.66 | 17.93 | 458,522 | 320,199 | 193,218 |
|  | LINKS | 6636 | 73 | 96.852 | 86.85 | 10.22 | 457,001 | 254,462 | 196,366 |
|  | npscarf | 6619 | 94 | 97.520 | 108.88 | 22.34 | 661,642 | 657,227 | 257,271 |
|  | SLR | 6665 | 71 | 97.229 | 97.60 | 11.64 | 656,348 | 498,471 | 270,402 |
| *Chr X*_1_SMRT | SSPACE-LR | 3351 | 176 | 97.675 | 4.08 | 2.69 | 4,252,643 | 1,041,385 | 809,815 |
|  | LINKS | 6744 | 100 | 97.551 | 4.12 | 1.81 | 1,905,621 | 189,701 | 183,461 |
|  | npscarf | 5396 | 157 | 97.767 | 94.14 | 4.24 | 11,142,909 | 3,610,237 | 2,325,939 |
|  | SLR | 5196 | 83 | 97.454 | 3.92 | 2.58 | 13,177,419 | 3,286,062 | 2,390,483 |

^1^ Missassemblies,^2^ mismatches per 100 kbp, ^3^ indels per 100 kbp

Table S2. Evaluation results based on Nanopore datasets

| Data set | Tool | Count | MA^1^ | Genome Fraction | Mismatches^2^ | Indels^3^ | Largest alignment | NG50 | NGA50 |
| --- | --- | --- | --- | --- | --- | --- | --- | --- | --- |
| *E.coli_*1_ONT | SSPACE-LR | 106 | 6 | 99.640 | 2.21 | 1.71 | 1,350,083 | 1,341,884 | 1,175,277 |
|  | LINKS | 109 | 5 | 99.361 | 1.60 | 1.21 | 1,202,180 | 1,094,000 | 693,473 |
|  | npscarf | 72 | 6 | 99.807 | 2.72 | 2.20 | 2,295,163 | 4,635,772 | 1,672,119 |
|  | SLR | 75 | 4 | 99.944 | 1.85 | 3.38 | 2,927,247 | 4,678,549 | 2,927,247 |
| *E.coli*_2_ ONT | SSPACE-LR | 124 | 13 | 99.420 | 2.60 | 0.93 | 1,348,829 | 1,357,410 | 607,813 |
|  | LINKS | 130 | 9 | 99.366 | 2.36 | 0.39 | 852,792 | 693,259 | 444,737 |
|  | npscarf | 117 | 11 | 99.545 | 6.23 | 3.31 | 1,552,547 | 1,746,790 | 687,270 |
|  | SLR | 114 | 9 | 99.695 | 3.13 | 1.90 | 1,593,671 | 1,749,414 | 733,062 |
| *S.cerevisiae*_1_ ONT | SSPACE-LR | 2044 | 215 | 97.318 | 89.65 | 14.85 | 457,344 | 247,071 | 190,419 |
|  | LINKS | 2846 | 52 | 96.734 | 78.91 | 9.91 | 455,351 | 261,536 | 231,114 |
|  | npscarf | 2755 | 83 | 97.214 | 102.09 | 17.53 | 949,878 | 749,986 | 374,986 |
|  | SLR | 2823 | 46 | 96.997 | 82.45 | 11.87 | 1,068,398 | 669,916 | 374,835 |
| *S.cerevisiae*_2_ ONT | SSPACE-LR | 5134 | 297 | 97.366 | 90.83 | 16.75 | 465,668 | 366,246 | 176,096 |
|  | LINKS | 6697 | 69 | 96.785 | 84.89 | 9.78 | 457,640 | 223,875 | 190,743 |
|  | npscarf | 6625 | 78 | 97.275 | 105.07 | 16.47 | 575,056 | 578,365 | 323,202 |
|  | SLR | 6671 | 68 | 97.086 | 86.07 | 11.17 | 652,163 | 546,046 | 270,362 |

^1^ Missassemblies,^2^ mismatches per 100 kbp, ^3^ indels per 100 kbp

**4. Different values of L_ca_ for scaffolding**

SLR considers the contig whose length shorter than *L_ca_* as ambiguous contig, and these contigs also can be inserted into scaffolds produced by unique contigs. *L_ca_* is 3000 in default, and it can be set by users. We set different values of *L_ca_* to validate its influence, and the results are shown in the following Table S3 and Table S4.

Table S3. Different values of *L_ca_* of SLR using SMRT datasets

| Data set | *L_ca_* | Count | MA^1^ | Genome Fraction | Mismatches^2^ | Indels^3^ | Largest alignment | NGA50 |
| --- | --- | --- | --- | --- | --- | --- | --- | --- |
| *E.coli_*1_SMRT | 1000 | 85 | 4 | 99.852 | 2.35 | 3.82 | 857,292 | 656,592 |
|  | 2000 | 84 | 4 | 99.873 | 2.42 | 3.88 | 85,7291 | 656,585 |
|  | 3000 | 83 | 4 | 99.873 | 2.07 | 3.39 | 1,011,630 | 723,879 |
|  | 4000 | 82 | 4 | 99.824 | 2.91 | 3.28 | 1,002,999 | 723,879 |
| *E.coli*_2_SMRT | 1000 | 119 | 11 | 99.596 | 3.16 | 1.49 | 855,371 | 566,538 |
|  | 2000 | 118 | 11 | 99.596 | 3.16 | 1.51 | 855,370 | 566,538 |
|  | 3000 | 118 | 10 | 99.598 | 2.99 | 1.54 | 855,361 | 565,864 |
|  | 4000 | 118 | 10 | 99.540 | 2.81 | 1.47 | 855,348 | 565,864 |
| *S.cerevisiae*_1_SMRT | 1000 | 2784 | 53 | 97.249 | 95.79 | 12.69 | 1,079,981 | 374,743 |
|  | 2000 | 2784 | 53 | 97.249 | 95.79 | 12.69 | 1,079,980 | 374,744 |
|  | 3000 | 2784 | 52 | 97.250 | 95.79 | 12.71 | 1,079,981 | 374,744 |
|  | 4000 | 2784 | 52 | 97.250 | 95.78 | 12.67 | 1,079,984 | 374,744 |
| *S.cerevisiae*_2_SMRT | 1000 | 6664 | 72 | 97.245 | 98.12 | 11.69 | 656,348 | 270,402 |
|  | 2000 | 6664 | 72 | 97.245 | 98.12 | 11.67 | 656,348 | 270,402 |
|  | 3000 | 6665 | 71 | 97.229 | 97.60 | 11.64 | 656,348 | 270,402 |
|  | 4000 | 6666 | 71 | 97.231 | 97.55 | 11.58 | 656,348 | 270,401 |
| *Chr X*_1_SMRT | 1000 | 5218 | 80 | 97.459 | 3.91 | 2.60 | 13,177,440 | 2,165,615 |
|  | 2000 | 5220 | 80 | 97.457 | 3.91 | 2.60 | 13,177,431 | 2,165,616 |
|  | 3000 | 5196 | 83 | 97.454 | 3.92 | 2.58 | 13,177,419 | 2,390,483 |
|  | 4000 | 5198 | 83 | 97.452 | 3.82 | 2.58 | 13,177,428 | 2,390,459 |

Table S4. Different values of *L_ca_* of SLR using Nanopore datasets

| Data set | *L_ca_* | Count | MA^1^ | Genome Fraction | Mismatches^2^ | Indels^3^ | Largest alignment | NA50 | NGA50 |
| --- | --- | --- | --- | --- | --- | --- | --- | --- | --- |
| *E.coli_*1_ONT | 1000 | 75 | 4 | 99.944 | 1.85 | 3.38 | 2,927,164 | 2,927,164 | 2,927,164 |
|  | 2000 | 75 | 4 | 99.944 | 1.85 | 3.38 | 2,927,164 | 2,927,164 | 2,927,164 |
|  | 3000 | 75 | 4 | 99.944 | 1.85 | 3.38 | 2,927,247 | 2,927,247 | 2,927,247 |
|  | 4000 | 75 | 4 | 99.944 | 1.81 | 3.41 | 2,927,163 | 2,927,163 | 2,927,163 |
| *E.coli*_2_ ONT | 1000 | 114 | 9 | 99.695 | 3.13 | 1.90 | 1,593,670 | 698,655 | 733,074 |
|  | 2000 | 114 | 9 | 99.695 | 3.13 | 1.90 | 1,593,670 | 698,655 | 733,073 |
|  | 3000 | 114 | 9 | 99.695 | 3.13 | 1.90 | 1,593,671 | 698,655 | 733,062 |
|  | 4000 | 114 | 9 | 99.695 | 3.13 | 1.90 | 1,593,669 | 698,655 | 733,062 |
| *S.cerevisiae*_1_ ONT | 1000 | 2823 | 46 | 97.001 | 82.46 | 11.87 | 1,068,395 | 344,909 | 374,836 |
|  | 2000 | 2823 | 46 | 96.997 | 82.45 | 11.87 | 1,068,398 | 344,909 | 374,836 |
|  | 3000 | 2823 | 46 | 96.997 | 82.45 | 11.87 | 1,068,398 | 344,909 | 374,835 |
|  | 4000 | 2822 | 46 | 97.001 | 82.46 | 11.93 | 1,068,399 | 344,909 | 374,835 |
| *S.cerevisiae*_2_ ONT | 1000 | 6671 | 68 | 97.086 | 86.07 | 11.17 | 652,163 | 246,305 | 270,359 |
|  | 2000 | 6671 | 68 | 97.086 | 86.07 | 11.17 | 652,163 | 246,305 | 270,362 |
|  | 3000 | 6671 | 68 | 97.086 | 86.07 | 11.17 | 652,163 | 246,306 | 270,362 |
|  | 4000 | 6671 | 73 | 97.084 | 86.47 | 11.02 | 652,163 | 240,235 | 255,875 |

**5. Scaffolding results about SLR1 and SLR2**

For verifying the effectiveness of the contig classification presented in this paper, we compare another version of SLR: SLR1, which does not use contig classification, and uses all contigs as unique contigs for scaffolding. The scaffolding results are shown in Table S5 and Table S6. For most datasets, the performance of SLR is better than SLR1 in terms of Misassemblies and NGA50.

For evaluating the effectiveness of edge weighting method in SLR, we design another version of SLR: SLR2. In SLR2, for an edge *e_ij_* in a scaffold graph *G*, its weight *w_ij_* is equal to the number of long reads aligning with *c_i_* and *c_j_* simultaneously. We compare the scaffolding results produced by SLR and SLR2. From the evaluation results shown in Table S5 and Table S6, we can see that the difference between SLR and SLR2 is small. In SLR, users can set a parameter to determine edge weighting method based on alignment length or read count.

Table S5. SLR, SLR1, and SLR2 based on SMRT datasets

| Data set | Tool | Count | MA^1^ | Genome Fraction | Mismatches^2^ | Indels^3^ | Largest alignment | NG50 | NGA50 |
| --- | --- | --- | --- | --- | --- | --- | --- | --- | --- |
| *E.coli_*1_SMRT | SLR | 83 | 4 | 99.873 | 2.07 | 3.39 | 1,011,630 | 723,879 | 723,879 |
|  | SLR1 | 101 | 12 | 99.717 | 2.92 | 2.81 | 486,892 | 656569 | 295,999 |
|  | SLR2 | 86 | 4 | 99.871 | 3.15 | 3.95 | 723,876 | 628614 | 628,340 |
| *E.coli*_2_SMRT | SLR | 118 | 10 | 99.598 | 2.99 | 1.54 | 855,361 | 1,130,614 | 565,864 |
|  | SLR1 | 127 | 11 | 99.537 | 2.99 | 1.56 | 565,887 | 635369 | 197,175 |
|  | SLR2 | 120 | 12 | 99.577 | 5.02 | 1.49 | 855,361 | 653055 | 457,758 |
| *S.cerevisiae*_1_SMRT | SLR | 2784 | 52 | 97.25 | 95.79 | 12.71 | 1,079,981 | 736,056 | 374,744 |
|  | SLR1 | 2723 | 57 | 96.77 | 85.25 | 12.46 | 526,940 | 418033 | 232,712 |
|  | SLR2 | 2783 | 50 | 97.330 | 93.37 | 12.54 | 1,079,981 | 736,056 | 374,744 |
| *S.cerevisiae*_2_SMRT | SLR | 6665 | 71 | 97.229 | 97.60 | 11.64 | 656,348 | 498,471 | 270,402 |
|  | SLR1 | 6532 | 67 | 97.041 | 89.87 | 12.27 | 468,888 | 546669 | 201,922 |
|  | SLR2 | 6660 | 71 | 97.262 | 97.75 | 11.70 | 656,348 | 498,471 | 270,402 |
| *ChrX* _1_SMRT | SLR | 5196 | 83 | 97.454 | 3.92 | 2.58 | 13,177,419 | 3,286,062 | 2,390,483 |
|  | SLR1 | 5188 | 82 | 97.476 | 4.07 | 2.61 | 13,177,440 | 2,583,658 | 2,165,615 |
|  | SLR2 | 5196 | 84 | 97.449 | 4.02 | 2.59 | 13,177,419 | 3,296,062 | 2,390,483 |

^1^ Missassemblies,^2^ mismatches per 100 kbp, ^3^ indels per 100 kbp

Table S6. SLR, SLR1, and SLR2 based on Nanopore datasets

| Data set | Tool | Count | MA^1^ | Genome Fraction | Mismatches^2^ | Indels^3^ | Largest alignment | NG50 | NGA50 |
| --- | --- | --- | --- | --- | --- | --- | --- | --- | --- |
| *E.coli_*1_ONT | SLR | 75 | 4 | 99.944 | 1.85 | 3.38 | 2,927,247 | 4,678,549 | 2,927,247 |
|  | SLR1 | 105 | 8 | 99.864 | 2.72 | 2.98 | 806,502 | 1,426,114 | 674,408 |
|  | SLR2 | 75 | 4 | 99.944 | 1.85 | 3.38 | 2,567,139 | 4,734,406 | 2,567,139 |
| *E.coli*_2_ ONT | SLR | 114 | 9 | 99.695 | 3.13 | 1.90 | 1,593,671 | 1,749,414 | 733,062 |
|  | SLR1 | 126 | 14 | 99.517 | 2.64 | 1.02 | 1,015,798 | 1,012,842 | 361,345 |
|  | SLR2 | 114 | 9 | 99.695 | 3.13 | 1.90 | 1,593,671 | 1,749,414 | 733,062 |
| *S.cerevisiae*_1_ ONT | SLR | 2823 | 46 | 96.997 | 82.45 | 11.87 | 1,068,398 | 669,916 | 374,835 |
|  | SLR1 | 2740 | 66 | 97.063 | 83.86 | 12.29 | 556211 | 445,225 | 244,417 |
|  | SLR2 | 2822 | 47 | 97.019 | 79.88 | 11.59 | 1,068,398 | 734,803 | 374,835 |
| *S.cerevisiae*_2_ ONT | SLR | 6671 | 68 | 97.086 | 86.07 | 11.17 | 652,163 | 546,046 | 270,362 |
|  | SLR1 | 6477 | 85 | 97.069 | 88.50 | 12.05 | 533,351 | 546,046 | 201,066 |
|  | SLR2 | 6671 | 68 | 97.060 | 86.09 | 11.17 | 652,163 | 546,046 | 270,362 |

^1^ Missassemblies,^2^ mismatches per 100 kbp, ^3^ indels per 100 kbp

**6. SSPACE-LR and LINKS combined with contig classification method**

SSPACE-LR-CC represents the method based on SSPACE-LR combined with contig classification. LINKS-CC represents the method based on LINKS combined with contig classification.

Table S7. SSPACE-LR, SSPACEE-LR-CC, LINKS and LINKS-CC based on SMRT datasets

| Data set | Tool | Count | MA^1^ | Genome Fraction | Mismatches^2^ | Indels^3^ | Largest alignment | NG50 | NGA50 |
| --- | --- | --- | --- | --- | --- | --- | --- | --- | --- |
| *E.coli_*1_SMRT | SSPACE-LR | 102 | 10 | 99.550 | 2.12 | 1.21 | 952,193 | 1,084,156 | 538,319 |
|  | SSPACE-LR-CC | 89 | 11 | 99.702 | 2.55 | 3.11 | 716,930 | 658,199 | 436,445 |
|  | LINKS | 111 | 6 | 99.351 | 1.71 | 1.02 | 855,618 | 506,227 | 445,266 |
|  | LINKS-CC | 94 | 5 | 99.751 | 2.63 | 3.09 | 857,835 | 697,648 | 695,951 |
| *E.coli*_2_SMRT | SSPACE-LR | 124 | 12 | 99.364 | 2.25 | 0.54 | 693,087 | 661,478 | 361,326 |
|  | SSPACE-LR-CC | 118 | 11 | 99.581 | 2.88 | 1.15 | 698,397 | 696,716 | 659,446 |
|  | LINKS | 127 | 9 | 99.354 | 2.23 | 0.41 | 855,943 | 654,326 | 458,412 |
|  | LINKS-CC | 122 | 11 | 99.535 | 2.53 | 1.06 | 698,397 | 696,130 | 460,606 |
| *S.cerevisiae*_1_SMRT | SSPACE-LR | 2237 | 101 | 97.209 | 84.61 | 13.51 | 576,123 | 245,251 | 200,813 |
|  | SSPACE-LR-CC | 2824 | 67 | 96.939 | 89.42 | 11.37 | 1,071,612 | 1,091,987 | 327,875 |
|  | LINKS | 2800 | 54 | 96.734 | 78.77 | 10.25 | 741,980 | 262,335 | 203,642 |
|  | LINKS-CC | 2874 | 53 | 96.826 | 83.07 | 10.92 | 527,741 | 338,121 | 244,387 |
| *S.cerevisiae*_2_SMRT | SSPACE-LR | 5260 | 155 | 97.421 | 95.66 | 17.93 | 458,522 | 320,199 | 193,218 |
|  | SSPACE-LR-CC | 6676 | 91 | 96.959 | 90.16 | 11.16 | 542,046 | 1,290,194 | 261,180 |
|  | LINKS | 6636 | 73 | 96.852 | 86.85 | 10.22 | 457,001 | 254,462 | 196,366 |
|  | LINKS-CC | 6698 | 80 | 96.803 | 86.98 | 10.23 | 467,266 | 319,566 | 196,509 |
| *Chr X*_1_SMRT | SSPACE-LR | 3351 | 176 | 97.675 | 4.08 | 2.69 | 4,252,643 | 1,041,385 | 809,815 |
|  | SSPACE-LR-CC | 5450 | 114 | 97.299 | 2.60 | 2.28 | 13,306,6207 | 6,045,072 | 3,062,900 |
|  | LINKS | 6744 | 100 | 97.551 | 4.12 | 1.81 | 1,905,621 | 189,701 | 183,461 |
|  | LINKS-CC | 6098 | 148 | 97.588 | 4.58 | 2.28 | 2,010,404 | 503,213 | 446,315 |

^1^ Missassemblies,^2^ mismatches per 100 kbp, ^3^ indels per 100 kbp

Table S8. SSPACE-LR, SSPACEE-LR-CC, LINKS and LINKS-CC based on Nanopore datasets

| Data set | Tool | Count | MA^1^ | Genome Fraction | Mismatches^2^ | Indels^3^ | Largest alignment | NG50 | NGA50 |
| --- | --- | --- | --- | --- | --- | --- | --- | --- | --- |
| *E.coli_*1_ONT | SSPACE-LR | 106 | 6 | 99.640 | 2.21 | 1.71 | 1,350,083 | 1,341,884 | 1,175,277 |
|  | SSPACE-LR-CC | 120 | 8 | 99.360 | 1.26 | 1.19 | 1,593,972 | 3,687,370 | 1,269,078 |
|  | LINKS | 109 | 5 | 99.361 | 1.60 | 1.21 | 1,202,180 | 1,094,000 | 693,473 |
|  | LINKS-CC | 83 | 6 | 99.833 | 3.32 | 3.09 | 1,930,198 | 1,362,192 | 1,123,503 |
| *E.coli*_2_ ONT | SSPACE-LR | 124 | 13 | 99.420 | 2.60 | 0.93 | 1,348,829 | 1,357,410 | 607,813 |
|  | SSPACE-LR-CC | 112 | 14 | 99.495 | 2.25 | 1.36 | 1,481,236 | 2,509,849 | 697,751 |
|  | LINKS | 130 | 9 | 99.366 | 2.36 | 0.39 | 852,792 | 693,259 | 444,737 |
|  | LINKS-CC | 120 | 11 | 99.634 | 2.79 | 1.25 | 1,122,242 | 1,335,487 | 698,655 |
| *S.cerevisiae*_1_ ONT | SSPACE-LR | 2044 | 215 | 97.318 | 89.65 | 14.85 | 457,344 | 247,071 | 190,419 |
|  | SSPACE-LR-CC | 2864 | 82 | 96.806 | 80.09 | 10.86 | 591,140 | 530,152 | 224,381 |
|  | LINKS | 2846 | 52 | 96.734 | 78.91 | 9.91 | 455,351 | 261,536 | 231,114 |
|  | LINKS-CC | 2886 | 48 | 96.777 | 75.85 | 10.05 | 528,978 | 284,561 | 230,720 |
| *S.cerevisiae*_2_ ONT | SSPACE-LR | 5134 | 297 | 97.366 | 90.83 | 16.75 | 465,668 | 366,246 | 176,096 |
|  | SSPACE-LR-CC | 6693 | 89 | 96.848 | 85.21 | 10.48 | 467,342 | 451,245 | 182,246 |
|  | LINKS | 6697 | 69 | 96.785 | 84.89 | 9.78 | 457,640 | 223,875 | 190,743 |
|  | LINKS-CC | 6718 | 74 | 96.864 | 84.55 | 9.93 | 467,318 | 246,232 | 190,743 |

^1^ Missassemblies,^2^ mismatches per 100 kbp, ^3^ indels per 100 kbp

**7. Scaffolding results based on repeat-aware evaluation framework**

The evaluation framework firstly gives the numbers of total links, correct links and inferred links. Next, it gives three metrics: precision, recall and f1-score. The numbers of total links about four new contig sets are 432, 636, 3874 and 4430 respectively. We also use QUAST to evaluate the scaffolding result to obtain Misassemblies and NGA50.

As shown in Table S9 and Table S10, we can find that SSPACE-LR gets the best results in terms of F1-score. Meanwhile, the number of Misassemblies of SSPACE-LR is the largest than that of other tools. SLR gets the best value of NGA50. And SLR has small number of Misassemblies. For all datasets, the F1-score of SLR is better than other tools except SSPACE. Note that, for some datasets, the evaluation framework does not output evaluation result about npScarf, perhaps because npScarf makes sequence consensus between contigs and long reads.

Table S9. Scaffolding results based on new contig sets and SMRT datasets

| Data set | Tool | Correct links | Inferred links | Recall | Precision | F1-score | MA^1^ | NGA50 |
| --- | --- | --- | --- | --- | --- | --- | --- | --- |
| *E.coli_*1_SMRT_R | SSPACE-LR | 255 | 297 | **0.8585** | **0.5902** | **0.6995** | 15 | 421,951 |
|  | LINKS | 98 | 130 | 0.7538 | 0.2268 | 0.3487 | 5 | 178,580 |
|  | npScarf | * | * | * | * | * | 4 | 203,567 |
|  | SLR | 207 | 252 | 0.8214 | 0.4791 | 0.6052 | **2** | **594,423** |
| *E.coli*_2_SMRT_R | SSPACE-LR | 279 | 339 | **0.8230** | **0.4386** | **0.5723** | 10 | 570,077 |
|  | LINKS | 96 | 135 | 0.7111 | 0.1509 | 0.2490 | 5 | 171,786 |
|  | npScarf | 41 | 63 | 0.6507 | 0.0644 | 0.1173 | 2 | 206,839 |
|  | SLR | 236 | 298 | 0.7919 | 0.3710 | 0.5053 | **1** | **571,398** |
| *S.cerevisiae*_1_SMRT_R | SSPACE-LR | 1095 | 1300 | **0.8423** | **0.2826** | **0.4232** | 59 | 376,654 |
|  | LINKS | 574 | 757 | 0.7582 | 0.1481 | 0.2478 | 44 | 119,454 |
|  | npScarf | 143 | 301 | 0.4750 | 0.0369 | 0.0685 | 105 | 181,860 |
|  | SLR | 959 | 1178 | 0.8140 | 0.2475 | 0.3796 | **41** | **401,408** |
| *S.cerevisiae*_2_SMRT_R | SSPACE-LR | 1010 | 1278 | **0.7902** | **0.2279** | **0.3538** | 68 | 258,766 |
|  | LINKS | 569 | 730 | 0.7794 | 0.1284 | 0.2205 | 47 | 95,596 |
|  | npScarf | * | * | * | * | * | 75 | 217,096 |
|  | SLR | 902 | 1168 | 0.7722 | 0.2036 | 0.3222 | **43** | **315,737** |

^1^ Missassemblies; *Evaluation framework does not output evaluation result.

Table S10. Scaffolding results based on new contig sets and Nanopore datasets

| Data set | Tool | Correct links | Inferred links | Recall | Precision | F1-score | MA^1^ | NGA50 |
| --- | --- | --- | --- | --- | --- | --- | --- | --- |
| *E.coli_*1_ONT_R | SSPACE-LR | 246 | 289 | **0.8512** | **0.5694** | **0.6823** | 8 | 622,515 |
|  | LINKS | 89 | 125 | 0.7120 | 0.2060 | 0.3195 | **3** | 183,595 |
|  | npScarf | 4 | 21 | 0.1904 | 0.0092 | 0.0176 | 13 | 435,869 |
|  | SLR | 208 | 268 | 0.7761 | 0.4814 | 0.5942 | 5 | **2,558,407** |
| *E.coli*_2_ ONT_R | SSPACE-LR | 264 | 319 | **0.8275** | **0.4150** | **0.5528** | 3 | 847,197 |
|  | LINKS | 101 | 142 | 0.7112 | 0.1588 | 0.2596 | **2** | 258,502 |
|  | npScarf | 31 | 60 | 0.5166 | 0.0487 | 0.0890 | 11 | 390,689 |
|  | SLR | 188 | 280 | 0.6714 | 0.2955 | 0.4104 | 4 | **2,697,003** |
| *S.cerevisiae*_1_ ONT_R | SSPACE-LR | 1101 | 1344 | **0.8191** | **0.2842** | **0.4220** | 76 | 285,088 |
|  | LINKS | 559 | 724 | 0.7720 | 0.1442 | 0.2431 | 40 | 117,607 |
|  | npScarf | * | * | * | * | * | 77 | 197,875 |
|  | SLR | 530 | 822 | 0.6447 | 0.1368 | 0.2257 | **35** | **400,934** |
| *S.cerevisiae*_2_ ONT_R | SSPACE-LR | 1000 | 1294 | **0.7727** | **0.2257** | **0.3494** | 93 | 250,863 |
|  | LINKS | 564 | 706 | 0.7988 | 0.1273 | 0.2196 | **33** | 125,193 |
|  | npScarf | * | * | * | * | * | 69 | 231,212 |
|  | SLR | 520 | 843 | 0.6168 | 0.1173 | 0.1972 | 37 | **308,705** |

^1^ Missassemblies; *Evaluation framework does not output evaluation result.
